# Phosphorylation of Nrf2 at Multiple Sites by MAP Kinases Has a Limited Contribution in Modulating the Nrf2-Dependent Antioxidant Response

**DOI:** 10.1371/journal.pone.0006588

**Published:** 2009-08-11

**Authors:** Zheng Sun, Zheping Huang, Donna D. Zhang

**Affiliations:** 1 Department of Pharmacology & Toxicology, College of Pharmacy, University of Arizona, Tucson, Arizona, United States of America; 2 Department of Pediatrics, Division of Biology and Medicine, Brown University, Providence, Rhode Island, United States of America; University of Giessen Lung Center, Germany

## Abstract

The bZIP transcription factor Nrf2 has emerged as a pivotal regulator of intracellular redox homeostasis by controlling the expression of many endogenous antioxidants and phase II detoxification enzymes. Upon oxidative stress, Nrf2 is induced at protein levels through redox-sensitive modifications on cysteine residues of Keap1, a component of the E3 ubiquitin ligase that targets Nrf2 for ubiquitin-dependent degradation. The mitogen activated protein kinases (MAPKs) have previously been proposed to regulate Nrf2 in response to oxidative stress. However, the exact role of MAPKs and the underlying molecular mechanism remain poorly defined. Here we report the first evidence that Nrf2 is phosphorylated *in vivo* by MAPKs. We have identified multiple serine/threonine residues as major targets of MAPK-mediated phosphorylation. Combined alanine substitution on those residues leads to a moderate decrease in the transcriptional activity of Nrf2, most likely due to a slight reduction in its nuclear accumulation. More importantly, Nrf2 protein stability, primarily controlled by Keap1, is not altered by Nrf2 phosphorylation *in vivo*. These data indicate that direct phosphorylation of Nrf2 by MAPKs has limited contribution in modulating Nrf2 activity. We suggest that MAPKs regulate the Nrf2 signaling pathway mainly through indirect mechanisms.

## Introduction

Disruption of redox homeostasis is associated with the toxic effects of many environmental insults and pathogenesis of aging-related diseases such as cancer and neurodegenerative disorders [1], [2], [3], [4], [5]. Mammalian intracellular redox homeostasis is maintained mainly through transcriptional control of an array of antioxidative genes upon exposure to environmental insults that generate oxidative stress. As a key component of such a control system, the antioxidant response element (ARE) is a conservative *cis*-acting element found in the promoter regions of many genes encoding antioxidants and detoxification enzymes. The corresponding *trans-*acting factor for the ARE is the nuclear factor erythroid 2-related factor 2 (Nrf2), a protein belonging to the bZIP (basic-leucine zipper) transcription factor family [6], [7], [8].

The Nrf2-ARE system is responsible for both basal and inducible expression of many genes involved in antioxidant responses, such as NAD(P)H quinone oxidoreductase 1 (NQO1), heme oxygenase-1 (HO-1), glutathione S-transferase A1 (GSTA1, also known as GST-Ya in mouse), glutamate-cysteine ligase (also known as γ-glutamylcysteine synthetase, i.e. γGCS) modifier subunit (GCLM) and catalytic subunit (GCLC) [9], [10], [11], [12]. The importance of the Nrf2-ARE system is evidently demonstrated by findings that Nrf2 knockout mice display significantly increased sensitivity to chemical toxicants and carcinogens [13], [14].

Nrf2 is activated by a plethora of stress inducers, phyto-antioxidants, and pathological conditions. The current model of the molecular mechanism for Nrf2 activation is that oxidative stress modifies cysteine residues of Keap1, a component of the E3 ubiquitin ligase that targets Nrf2 for degradation, resulting in compromised E3 activity and enhanced Nrf2 protein stability. The subsequent elevation in Nrf2 protein levels leads to Nrf2 nuclear accumulation, increased ARE-Nrf2 binding, and transactivation of its downstream genes [15], [16], [17], [18], [19], [20], [21]. In addition to the primary mode of regulation on its protein stability controlled by Keap1, Nrf2 is also regulated at multiple levels such as nucleocytoplamic trafficking, acetylation, and phosphorylation [22], [23], [24], [25].

The mitogen-activated protein kinases (MAPKs) are a family of highly related kinases consisting of the extracellular signal-regulated protein kinases (ERKs), the *c-jun* N-terminal kinases (JNKs), the p38 kinases and other relatively less characterized kinases, all of which catalyze phosphorylation on either serine or threonine residues adjacent to a proline residue. MAPKs are activated in a cascade fashion, by MAP2Ks (also known as MKKs or MEKs) that are in turn activated by MAP3Ks (also known as MEKKs, including ASK1, TAK1, Rafs, etc.) [26]. The MAPK signaling system responds to diverse stimuli, including oxidative stress, and has been implicated in Nrf2 induction by many previous reports (Table 1, see Discussion) [24], [25]. However previous studies failed to address whether the observed effects of MAPKs on Nrf2 are through direct and specific phosphorylation events on Nrf2 or through indirect and less specific mechanisms. It is not even known whether or not MAPKs phosphorylate Nrf2 *in vivo* at all. Another question that remains unanswered is whether MAPKs affect Keap1-mediated control of Nrf2 protein stability *in vivo*. Therefore, the exact role of MAPKs in Nrf2 activation, as well as the underlying molecular mechanism, remains poorly understood.

**Table 1:** Summary of key findings by previous studies showing the involvement of MAPKs in Nrf2 activation.

Manipulation of MAPKs	Readout of Nrf2 activity	Changes of Nrf2 activity	Nrf2 inducers	Ref
PD98059 or DN ERK2	NQO1 enzyme, GSTA1 ARE-luc	30–60% decrease	tBHQ, SF	[42]
PD98059 and SB202190	GCS mRNA, ARE-luc, ARE binding	∼100% decrease	PDTC	[43]
MEKK1, TAK1, ASK1, MKK4/6, JNK1	GSTA1 ARE-luc	3–18 fold increase		[44]
p38	GSTA1 ARE-luc	30–40% decrease	tBHQ	[44]
SB203580, DN p38 or DN MKK3	NQO1 enzyme, GSTA1 ARE-luc	40–50% increase	BHA, SF, β-NF	[45]
SB202191 or DN p38α	HO-1 E1-luc	70–80% decrease	CdCl2	[46]
PD98059, DN JNK1/2 or DN ERK1/2	HO-1 E1-luc	no change	CdCl2	[46]
PD98059 and SB202191	nuclear Nrf2 protein	80–90% decrease	PDTC	[34]
JNK1	ARE-luc	∼6 fold increase	PEITC	[47]
DN JNK1	ARE-luc	20–70% decrease	PEITC	[47]
MEKK1, TAK1, ASK1, MEK1/5, ERK2/5	ARE-luc	1.5–5 fold increase		[35]
U0126 or ERK1 knockout	ARE-luc, ARE binding, Nrf2 protein	block induction	hyperoxia	[48]
SP600125 or SB203580	Nrf2 protein	block induction	gallic acid	[49]
Raf knockout	Nrf2 nulear entry	no change	curcumin	[50]
p38α, β, γ, δ	HO-1 promoter-luc	20–80% decrease	SF	[33]
U0126, DN ERK2 or DN JNK1	ARE-luc	20–60% decrease	BHA	[51]
MKK4 and JNK1	ARE-luc, HO-1	3 fold increase	PEITC	[52]
DN ERK2 or DN JNK1	ARE-luc	40–50% decrease	PEITC	[52]
PD98059 and SB203580	nuclear Nrf2 protein	block induction	4-HNE	[53]
U0126 or DN MEK	nuclear Nrf2 protein	block induction	PGG	[54]
PD169316 or p38 siRNA	HO-1 protein, nuclear Nrf2 protein	>50% decrease	PDT	[55]
PD98059 or SB203580	Nrf2 nulear entry	block induction	quercectin	[56]
PD98059 or U0126	nuclear Nrf2 protein, ARE-luc	∼60% decrease	D3T	[57]
U0126	Nrf2 nuclear entry, ARE-luc	block induction	Triphlorethol-A	[58]
PD98059	nuclear Nrf2 protein	block induction	eupatilin	[59]
PD98059	nuclear Nrf2, ARE-luc, ARE binding	block induction	MT-III	[60]
U0126, DN ERK1 or DN ERK2	HO-1 protein, ARE-luc	30–80% decrease	15d-PGJ2	[61]
U0126	nuclear Nrf2 protein	block induction	EGCG	[62]

Manipulation of MAPKs activities, by either using kinase inhibitors or genetic engineering of MAPKs by overexpressing wild-type or dominant-negative (DN) kinases, was used as the major approach to assess the role of MAPKs in Nrf2 activation. PD98059: MEK1 inhibitor; SB202190: p38 inhibitor; SB203580: p38 inhibitor; U0126 MEK1/2 inhibitor; SP6000125: JNK inihibitor; luc: luciferase reporter gene; PDTC: pyrrolidine dithiocarbamate; β-NF: beta-naphthoflavone; BHA: tert-butylhydroxyanisole; PEITC: phenethylisothiocyanates; PDT: photodynamic therapy; 4-HNE: 4-hydroxynonenal; D3T: 3H-1,2-dithiole-3-thione; PGG: 1,2,3,4,6-penta-O-galloyl-β-D-glucose; ISMC: ileal smooth muscle cell; MTIII: metallothionein-III; EGCG: Epigallocatechin gallate.

Here we report the first evidence indicating that Nrf2 is phosphorylated at several serine and threonine residues by MAPKs *in vivo*. Combined alanine substitution of those residues leads to only a very moderate decrease in the transcriptional activity of Nrf2, most likely due to a slight reduction in its nuclear accumulation. More importantly, Nrf2 protein stability, which is primarily controlled by Keap1, is not altered *in vivo* by Nrf2 phosphorylation. These data indicate that direct phosphorylation of Nrf2 by MAPKs has limited contribution in modulating the Nrf2 signaling pathway. We propose that MAPKs regulate Nrf2 mainly through indirect mechanisms.

## Materials and Methods

### Recombinant DNA molecules

Plasmids expressing JNK1/2, ERK2, p38, MEKK3 and MEKK4 were generous gifts from Dr. Richard R. Vaillancourt at University of Arizona. Plasmids for HA-Nrf2 and Keap1-CBD proteins have been previously described [27]. The Nrf2 mutants were generated by site-directed mutagenesis using the PCR and Dpn1 based method as described before [28]. The human NQO1-ARE TATA-Inr luciferase reporter plasmid and the mouse GSTA1-ARE TATA-Inr luciferase reporter plasmid were reported previously [28], [29]. Renilla luciferase plasmid pGL4.74[hRluc/TK] was purchased from Promega.

### Cell culture, transfection and chemicals

HEK293T, MDA-MB-231 and NIH3T3 cells were purchased from ATCC. Cells were maintained in either Dulbecco's modified Eagle's medium (DMEM) or Eagle's minimal essential medium (MEM) in the presence of 10% fetal bovine serum (FBS). Transfections were performed with Lipofectamine Plus (Gibco BRL) according to the manufacturer's instructions.

### Immunoblot, immunoprecipitation, and immunofluorescence analysis

Antibodies against the HA epitope (Santa Cruz), chitin-binding domain (CBD) (New England Biolab), α-tubulin (Santa Cruz), and phosphorylated serine/threonine followed by a proline (pS/TP) (Abcam) were purchased from commercial sources. For detection of protein expression in total cell lysates, cells were lysed in sample buffer (50 mM Tris-HCl [pH 6.8], 2% SDS, 10% Glycerol, 100 mM DTT, 0.1% bromophenol blue) 48 hours following transfection. For immunoprecipitation assays, cells were lysed in RIPA buffer (10 mM sodium phosphate pH [8.0], 150 mM NaCl, 1% Triton X-100, 1% sodium deoxycholate, 0.1% SDS) containing 1 mM dithiothreitol (DTT), 1 mM phenylmethylsulfonyl fluoride (PMSF), and a protease inhibitor cocktail (Sigma). Cell lysates were pre-cleared with protein A beads and incubated with HA antibody-conjugated beads (Santa Cruz) or chitin beads (New England Biolab) overnight at 4°C. After washing three times with RIPA buffer, immunoprecipitated complexes were eluted in sample buffer by boiling for 5 minutes, electrophoresed through SDS-polyacrylamide gels, and subjected to immunoblot analysis. Indirect immunofluorescence analysis was performed as described before using anti-HA primary antibodies [28].

### *In vivo* phosphorylation of Nrf2

To detect phosphorylated Nrf2 *in vivo* by immunoblot, transfected cells were lysed by boiling in a buffer containing 2% SDS, 150 mM NaCl, 10 mM Tris-HCl and 1 mM DTT. This rapid lysis procedure inactivated cellular phosphatases and therefore preserved phosphorylated-Nrf2 conjugates present in cells prior to lysis. These lysates were then diluted five-fold in buffer lacking SDS, followed by immunoprecipitation with HA antibody-conjugated beads.

### Mass spectrometry

Mass spectrometry analysis was performed by the Harvard Taplin mass spectrometry core facility. Briefly, immunoprecipitated proteins were visualized by Coomassie staining and recoverd from the gel. Excised gel bands were cut into approximately 1 mm^3^ pieces. Gel pieces were then subjected to a modified in-gel trypsin digestion procedure [30], followed by washing and dehydration with acetonitrile for 10 min. After removal of acetonitrile, gel pieces were completely dried in a speed-vac, and rehydrated with 50 mM ammonium bicarbonate solution containing 12.5 ng/µl modified sequencing-grade trypsin (Promega, Madison, WI) at 4°C. After 45 min., the excess trypsin solution was removed and replaced with 50 mM ammonium bicarbonate solution to just cover the gel pieces. Samples were then incubated at 37°C overnight. Peptides were later extracted by removing the ammonium bicarbonate solution, followed by one wash with a solution containing 50% acetonitrile and 5% acetic acid. The extracts were then dried in a speed-vac (∼1 hr). The samples were then reconstituted in 5–10 µl of HPLC solvent A (2.5% acetonitrile, 0.1% formic acid). A nano-scale reverse-phase HPLC capillary column was created by packing 5 µm C18 spherical silica beads into a fused silica capillary (100 µm inner diameter x∼12 cm length) with a flame-drawn tip. After equilibrating the column each sample was loaded via a Famos auto sampler (LC Packings, San Francisco CA) onto the column. A gradient was formed and peptides were eluted with increasing concentrations of solvent B (97.5% acetonitrile, 0.1% formic acid). As each peptide was eluted they were subjected to electrospray ionization and then they entered into an LTQ Orbitrap mass spectrometer (ThermoFinnigan, San Jose, CA). Eluting peptides were detected, isolated, and fragmented to produce a tandem mass spectrum of specific fragment ions for each peptide. Peptide sequences (and hence protein identity) were determined by matching protein or translated nucleotide databases with the acquired fragmentation pattern by the software program, Sequest (ThermoFinnigan, San Jose, CA).

### Reporter gene assay

A plasmid encoding renilla luciferase was included in all samples to control for transfection efficiency. Reporter assays were performed using the Dual-Luciferase reporter gene assay system (Promega). Nrf2 inducers *tert*-butylhydroquinone (tBHQ), sulforaphane (SF), sodium arsenite (As), and cadmium chloride (Cd) were purchased from Sigma (St. Louis, MO). Hydrogen peroxide (H2O2) was purchased from Mallinckrodt (Phillipsburg, NJ). Oridonin (Orid, also known as rubescensin A) was purchased from LKT Laboratories (St. Paul, MN). 15-Deoxy-Δ-12, 14-prostaglandin J2 (PGJ2) was a kind gift from Dr. John W. Regan at University of Arizona.

### qRT-PCR

The real-time RT-PCR analysis and primer/probe sequence information was previously described [28], [31], [32].

### Statistical test

Student's *t-* test was used to determine the significant difference between two samples from three assays in reporter gene analysis and qPCR analysis.

## Results

### Nrf2 is phosphorylated *in vivo* at multiple sites by MAPKs

**Figure 1:**
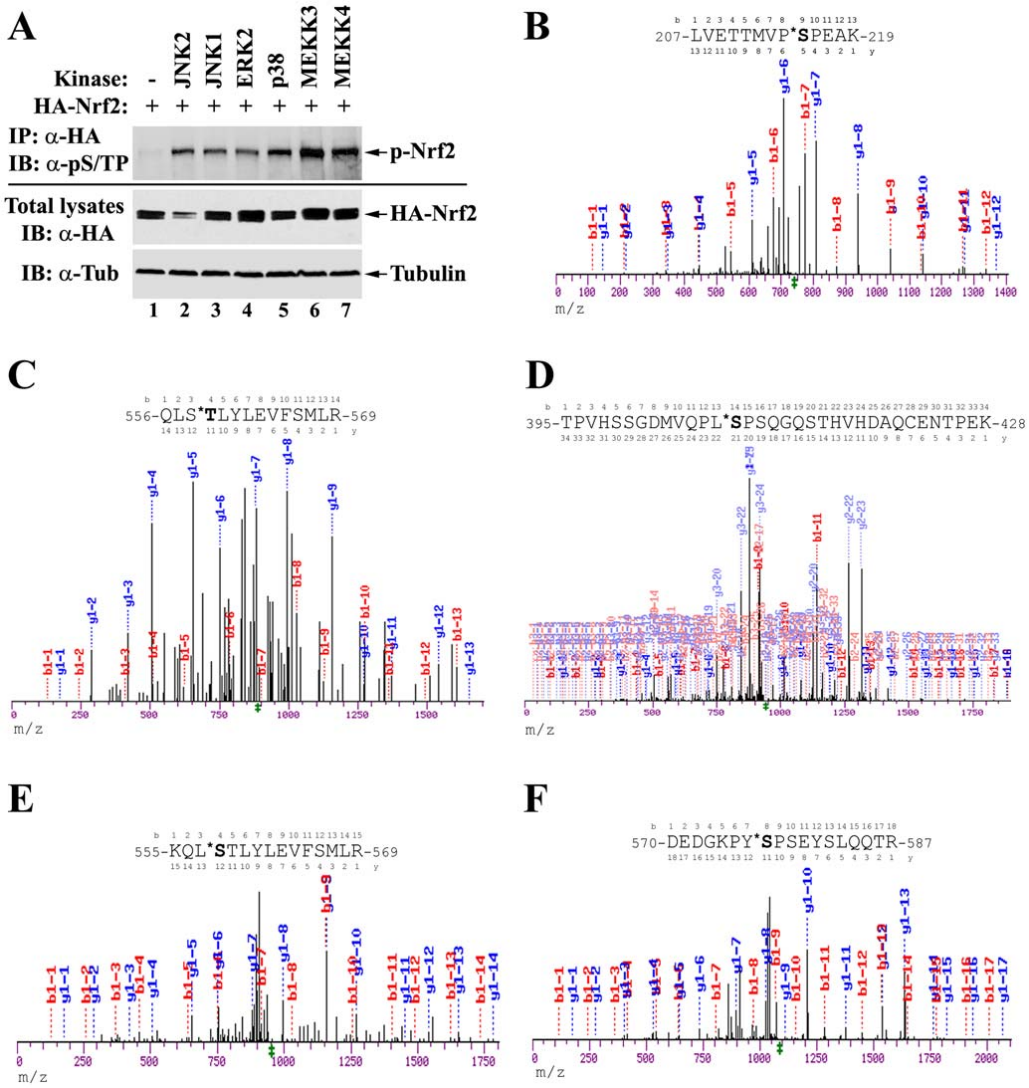
Nrf2 is phosphorylated *in vivo* at multiple sites by MAPKs. (A) HEK293T cells were cotransfected with expression vectors for HA-tagged Nrf2 and the indicated MAPKs. Cells were lysed in denaturing conditions. Cell lysates were diluted and immunoprecipitated with anti-HA antibodies. The immunoprecipitated protein was analyzed by immunoblot with antibodies specific for phosphorylated serine or threonine residues adjacent to a proline (pS/TP) (Abcam ab9344). p-Nrf2: phosphorylated Nrf2; Tub: tubulin. (B–F) Identification of distinct phosphorylation sites by tandem mass spectrometry analysis. Nrf2 proteins were purified through immunoprecipitation with anti-HA antibodies from HEK293T cells overexpressing HA-tagged Nrf2. The Nrf2 protein was size-separated by SDS-PAGE and visualized by Coomassie blue staining. The gel containing the Nrf2 protein was isolated and subjected to mass spectrometry analysis. m/z: mass to charge ratio.

Purified recombinant GST-Nrf2 protein has been shown to be phosphorylated by immunoprecipitated p38 MAPKs *in vitro* [33]. However it is not clear whether Nrf2 is a *bona fide* substrate of MAPKs *in vivo*. To address this question, HEK293T cells were cotransfected with expression vectors for HA-tagged Nrf2 and the indicated MAPKs (Figure 1A). Cell lysates were immunoprecipitated with anti-HA antibodies. The immunoprecipitates were analyzed by immunoblot using antibodies that recognize phosphorylated serine or threonine residue adjacent to a proline (pS/TP), the consensus motif phosphorylated by MAPKs. The phosphorylation of Nrf2 is significantly enhanced in the presence of overexpressed MAPKs or their upstream kinases (Figure 1A, compare lane 1 with others). The phosphorylation antibody is specific to MAPK consensus sites, as demonstrated by its failure to recognize non-MAPK phosphorylation sites such as IKK sites (Figure S2).

To identify the phosphorylation sites on Nrf2 by endogenous kinases, HEK293T cells were transfected with an expression vector for HA-tagged human Nrf2. Nrf2 proteins were purified by immunoprecipitation with anti-HA antibodies, separated by SDS-PAGE, and visualized by coomassie blue staining. The gel containing the Nrf2 protein was isolated and subjected to mass spectrometry analysis using LC-MS/MS. Five phosphorylated serine or threonine residues (S215, S408, S558, T559 and S577) were identified (Figure 1B–F). Three of them (S215, S408, S577) fit consensus sites for MAPKs, while the other two (S558 and S559) do not. All five sites were subsequently characterized, individually and combined.

### Abolishing Nrf2 phosphorylation by mutation causes a limited decrease in the transcriptional activity of Nrf2

**Figure 2:**
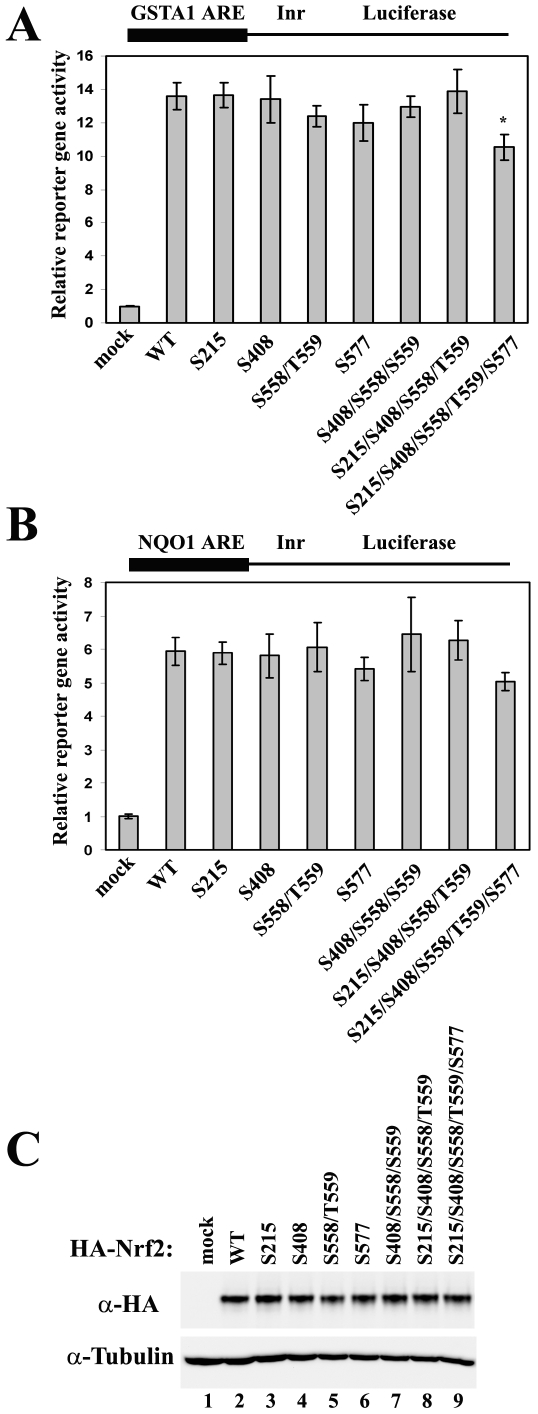
Combined alanine substitution on all phosphorylation sites causes only a moderate decrease in the transcriptional activity of Nrf2. (A) MDA-MB-231 cells were cotransfected with expression vectors for the GSTA1-ARE-dependent firefly luciferase, the TK-Renilla luciferase, and the indicated Nrf2 mutant. Luciferase reporter gene activities were analyzed using the Promega dual-luciferase reporter gene assay system. WT: wild-type; TK: thymidine kinase promoter; Inr: initiator. Relative luciferase activities and standard deviations were calculated from three independent assays. * p<0.05 when compared to WT. (B) Reporter gene assay was performed as described above using NQO1-ARE-dependent luciferase reporter gene construct in MDA-MB-231 cells. (C) Total cell lysates from one of the reporter gene assays were subjected to immunoblot analysis with anti-HA antibodies for detection of Nrf2.

Genetic approaches were taken to explore the function of phosphorylation on Nrf2. Single or combined alanine substitutions on the phosphorylation sites were constructed, and the transcriptional activity of the resultant Nrf2 mutants were tested in the ARE-dependent reporter gene assays using the ARE sequence taken from either the NQO1 gene promoter (Figure 2A) or the GSTA1 gene promoter (Figure 2B). In both cases, only the combined mutation of all five phosphorylation sites caused a slight decrease in Nrf2 transcriptional activity (Figure 2A and 2B). More importantly, none of the mutations, whether single or combined, caused obvious changes in Nrf2 protein levels (Figure 2C).

**Figure 3:**
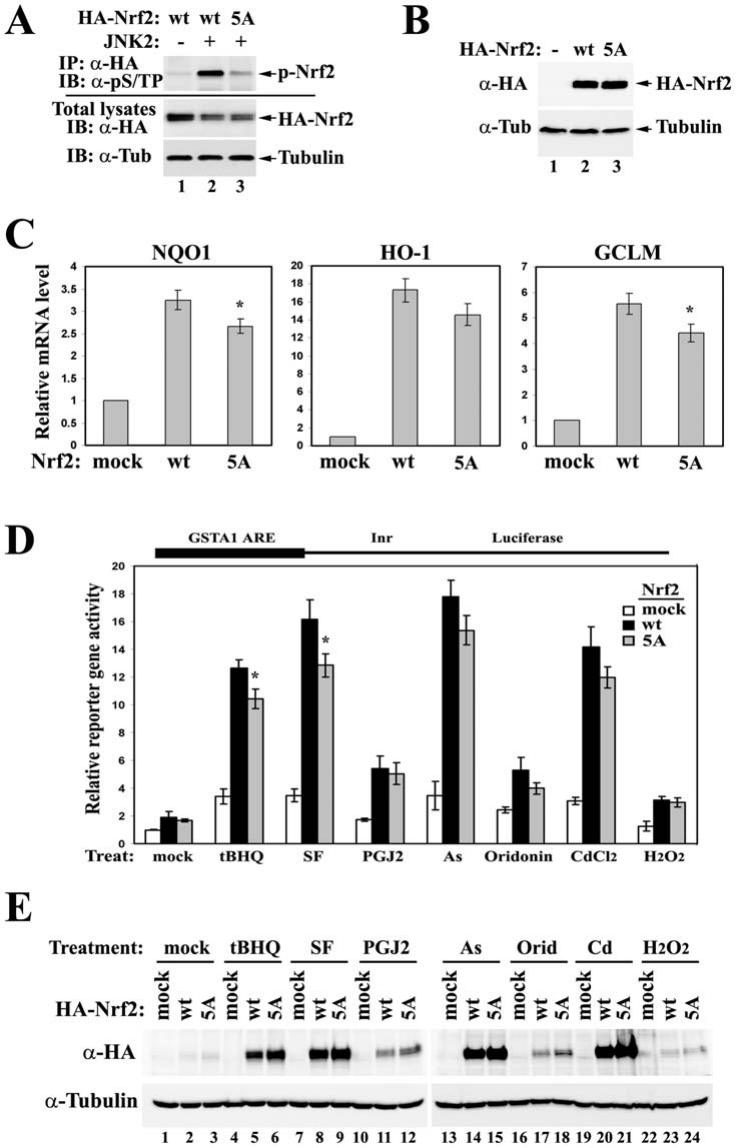
Genetic disruption of Nrf2 phosphorylation slightly attenuates Nrf2 activities without affecting its protein levels. (A) HEK293T cells were cotransfected with expression vectors for HA-tagged Nrf2 and JNK2. Cell lysates were immunoprecipitated with anti-HA antibodies. The immunoprecipitated protein was analyzed by immunoblot with antibodies specific for phosphorylated serine or threonine residue adjacent to a proline (pS/TP) (Abcam ab9344). wt: wild-type; 5A: the mutant with combined alanine substitution on all five phosphorylation sites. (B–C) HEK293T cells overexpressing either Nrf2-WT or Nrf2-5A were subjected to immunoblot (B) and qRT-PCR analysis (C). The error bars indicate the standard deviations calculated from triplicate samples. * p<0.05 when compared to wt. (D) MDA-MB-231 cells were cotransfected with expression vectors for the GSTA1-ARE-dependent luciferase reporter gene, Keap1, and the indicated Nrf2. Cells were treated with several Nrf2 inducers for 16 hrs prior to reporter gene analysis. tBHQ: tert-butylhydroquinone (50 µM); SF: sulforaphane (20 µM); PGJ2: 15-Deoxy-Δ-12,14-prostaglandin J2 (10 µM); As: sodium arsnite (20 µM); Orid: Oridonin (10 µM); Cd: cadmium chloride (20 µM); H_2_O_2_: hydrogen peroxide (500 µM). Error bars indicate standard deviations calculated from three independent assays. * p<0.05 when compared to wt in the same treatment group. (E) Total cell lysates from the above reporter gene assay were analyzed by immunoblot.

The mutant that has all five phosphorylation sites substituted by alanines was named “5A” and was subjected to subsequent analysis, since it seemed to have a slightly compromised ability in transactivating the ARE-dependent genes. Abolishment of Nrf2 phosphorylation in the Nrf2-5A mutant was confirmed by the *in vivo* phosphorylation assay (Figure 3A). All three MAPK consensus sites (S215, S408 and S577) seem to contribute to the overall phosphorylation level of Nrf2 in the presence of overexpressed MAPKs (Figure S1). If the antibody has no bias towards the context sequences around the consensus phosphorylation sites, S408 seems to be the most dominant site. None of the single mutations was able to abolish Nrf2 phosphorylation as well as the combined 5A mutation, suggesting a redundancy in phosphorylation sites (Figure S1). To further test the effects of phosphorylation on the transcriptional activity of Nrf2, mRNA expression of Nrf2 target genes was analyzed by qRT-PCR in HEK293T cells overexpressing either Nrf2-WT (wild-type) or Nrf2-5A mutant (Figure 3C). Loss of phosphorylation causes a minor or only moderate decrease in the expression of Nrf2 downstream gene NQO1, HO-1, and GCLM (Figure 3C), without affecting Nrf2 protein levels (Figure 3B and 3C).

### Phosphorylation of Nrf2 slightly increases the response of Nrf2 to several chemical inducers without affecting Keap1-mediated control of Nrf2 protein levels

To test whether phosphorylation of Nrf2 alters its behavior in response to different inducers, reporter gene analysis was performed on MDA-MB-231 cells overexpressing Keap1 and either Nrf2-WT or Nrf2-5A. Cells were treated with different Nrf2 inducers for 16 hrs prior to analysis. Altered response between Nrf2-WT and Nrf2-5A was only observed in the presence of relatively potent Nrf2 inducers (Figure 3D). The protein levels of Nrf2-WT and Nrf2-5A were increased to a similar extent in response to all inducers tested, suggesting that phosphorylation does not have any effect on Nrf2 protein stability that is primarily controlled by Keap1 (Figure 3E).

### Phosphorylation of Nrf2 by MAPKs does not affect the interaction between Nrf2 and Keap1 *in vivo*

**Figure 4:**
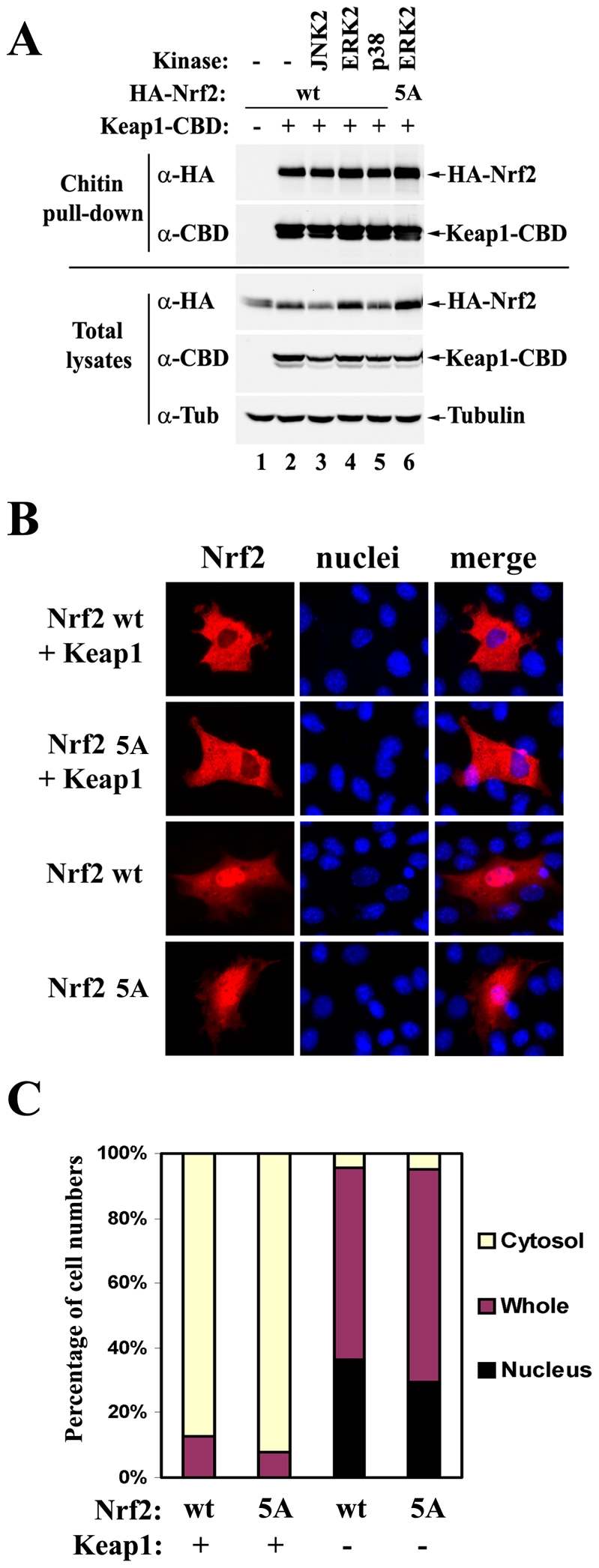
Phosphorylation of Nrf2 moderately enhances its nuclear accumulation without affecting the interaction between Nrf2 and Keap1. (A) HEK293T cells were cotransfected with expression vectors for the indicated MAPKs, HA-tagged Nrf2, and CBD-tagged Keap1. Cells were harvested and lysed in RIPA buffer. Cell lysates were pulled-down with chitin beads and analyzed by immunoblot with anti-HA and anti-CBD antibodies. CBD: chitin binding domain; wt: wild-type; 5A: mutant with combined alanine substitution on all five phosphorylation sites. (B) NIH3T3 cells were transfected with expression vectors for the indicated Nrf2 protein and Keap1. The subcellular localization of Nrf2 was determined by indirect immunofluorescence analysis with anti-HA antibodies. (C) Quantification analysis of the immunofluorescence assay. At least 100 positively stained cells were examined by fluorescence microscopy. Percentages of cells in which Nrf2 was localized predominantly in the cytosol, the whole cell, or the nucleus were presented as a bar graph.

Keum et al. have reported that phosphorylation of Nrf2 by p38 MAPKs increased the interaction between Nrf2 and Keap1 in an *in vitro* GST-pulldown experiment [33]. To test whether similar effects can be observed *in vivo*, HEK293T cells were co-transfected with an expression vector for either HA-tagged Nrf2-WT or Nrf2-5A, along with expression vectors for CBD-tagged Keap1, and the indicated MAPK. Cell lysates were pulled-down with chitin beads and the precipitated protein complexes were analyzed by immunoblot with anti-HA and anti-CBD antibodies (Figure 4A). The interaction between Nrf2 and Keap1 was not affected by the phosphorylation status of Nrf2, consistent with the finding that phoshorylation of Nrf2 does not affect its protein stability (Figure 3E).

### Phosphorylation of Nrf2 moderately enhances its nuclear accumulation

Nuclear translocation of Nrf2 protein is essential for Nrf2 to transactivate its downstream genes. To test whether phosphorylation of Nrf2 alters its nuclear accumulation, NIH3T3 cells were transfected with the expression vector for Nrf2-WT or Nrf2-5A in the presence or absence of the expression vector for Keap1 to mimic basal or induced conditions, respectively. The subcellular localization of Nrf2 was determined by indirect immunofluorescence analysis with anti-HA antibodies. There was a slight decrease in nuclear accumulation of Nrf2-5A, compared to Nrf2-WT, in the absence of Keap1, as shown in representative pictures (Figure 4B) and quantification data (Figure 4C).

## Discussion

Many previous studies have shown that MAPKs play a role in the induction of Nrf2. Table 1 summarizes key findings of all previous studies that support the involvement of MAPKs in Nrf2 activation. Manipulation of the catalytic activity of MAPKs, either through pharmaceutical intervention (kinase inhibitors) or genetic engineering (overexpression of wild-type or dominant-negative kinases, siRNA-mediated knockdown, or gene knockout), coupled with a readout of Nrf2 activity (ARE-dependent luciferase reporter gene, or Nrf2 nuclear accumulation), was used as the major approach to address the potential role of MAPKs on Nrf2 (Table 1). All three well-characterized categories of MAPKs (ERKs, JNKs, and p38) have been implicated in modulating Nrf2, with some contradictory results among different groups. Since MAPKs regulate bewilderingly diverse cellular programs, it is not clear whether the observed effects of MAPKs on Nrf2 are highly specific or just bystander effects. It remains elusive whether MAPKs regulate Nrf2 through direct phosphorylation of Nrf2 or through indirect mechanisms. Furthermore, it is not known whether or not MAPKs phosphorylate Nrf2 *in vivo* at all. Another key question that remains open is whether MAPKs affect Nrf2 protein stability that is primarily controlled by Keap1. Therefore, the exact role of MAPKs in Nrf2 activation, as well as the underlying molecular mechanism, remains poorly understood.

In this study, we report the first evidence that Nrf2 is phosphorylated by MAPKs *in vivo*. We have identified the major phosphorylation sites and used site-mutagenesis to decipher the function of direct phosphorylation of Nrf2 by MAPKs. As demonstrated by data obtained from ARE-dependent luciferase reporter gene assay and qRT-PCR analysis, a loss of Nrf2 phosphorylation caused only moderate changes in the transcriptional activity of Nrf2 (Figure 2 and 3). The Keap1-mediated control of Nrf2 protein levels was not affected by Nrf2 phosphorylation *in vivo* under both basal and induced conditions (Figure 3E), nor was the interaction between Nrf2 and Keap1 (Figure 4A). However, the nuclear accumulation of Nrf2 was slightly enhanced by its phosphorylation (Figure 4B and 4C). We concluded that direct phosphorylation of Nrf2 by MAPKs has a limited contribution in regulating the Nrf2-dependent antioxidant responses.

We are aware of the fact that our mass spectrometry analysis may not have identified all phosphorylated residues on Nrf2. There might be signal-induced Nrf2 phosphorylation on specific sites that were missed in the current identification procedure whereby overexpressed Nrf2 protein itself was purified from HEK293T cells under basal conditions followed by LC-MS/MS. However, the fact that the Nrf2-5A mutant has significantly decreased phosphorylation levels compared to wild-type Nrf2 in the presence of overexpressed JNK2 suggests that these five residues are the major targets of phosphorylation *in vivo* (Figure 3A). Neither inducible ARE-dependent transcription nor inducible Nrf2 protein levels was dramatically altered by combined mutations on the identified phosphorylation sites in the presence of several Nrf2 inducers such as tBHQ, sulforaphane, PGJ2, arsenite or cadmium, suggesting that phosphorylation at these sites has limited contribution in regulating inducible Nrf2 signaling (Figure 3D and 3F). Additionally, in an experiment without using mutations, overexpression of MAPKs failed to cause any observable effect on the interaction between wild-type Nrf2 and Keap1 (Figure 4A, lane 1–5), although Nrf2 phosphorylation was significantly enhanced under the same conditions (Figure 1A). Collectively, these data suggest that direct phosphorylation of Nrf2 plays a limited role in regulating Nrf2 activity.

Consistent with our data, Zipper and Mulcahy showed that mutational disruption of six MAPK consensus sites in Nrf2 (including S215, S408, S577 that were also characterized in this study) failed to significantly change the GCLM ARE-dependent reporter gene activities in the absence or presence of pyrrolidine dithiocarbamate (PDTC), an Nrf2 inducer [34]. Here we provide additional evidence that mutational disruption of these sites does not dramatically alter the endogenous mRNA levels of NQO1, HO-1 or GCLM (Figure 3C), excluding possibilities that phosphorylation might interfere with molecular events that are specific to native chromosomal architectures. In addition, we showed that neither Nrf2 protein levels nor ARE-dependent luciferase activities were dramatically altered by the combined mutation in cells either left untreated or treated with divergent Nrf2 inducers, such as tBHQ, sulforaphane, PGJ2, arsnite, oridonin, cadmium and H_2_O_2_ (Figure 3E), making it a much less attractive hypothesis that phosphorylation might be involved in the action of some particular Nrf2 inducers, if not all. Furthermore, we performed a co-immunoprecipitation assay and directly demonstrated that the physical interaction between Nrf2 and Keap1 was not altered *in vivo* in the presence of overexpressed MAPKs or with Nrf2 phosphorylation-deficient mutants (Figure 4A). Another previous report from Kong and colleagues also showed that site-directed mutagenesis of Nrf2 at several MAPK consensus sites did not affect the transactivation activity of Nrf2, although these sites do not overlap with the sites characterized in our present study [35]. Taken together, MAPK-mediated regulation of the Nrf2 signaling pathway is not likely to be through direct phosphorylation of Nrf2.

One possible indirect mechanism is translational control on Nrf2 protein synthesis by MAPKs. MAPKs have been shown to modulate mTOR signaling pathways in controlling eukaryotic initiation factor 4 (eIF4) complex assembly, a critical step in Cap-dependent translational initiation, through inhibiting tuberous sclerosis complex 2 (TSC2) and activating 90-kD ribosomal protein S6 kinases (RSKs) [36], [37]. MAPKs also promote phosphorylation of eIF4E, eIF4B-BP1, and translation elongation factor 2 kinase (eEF2 kinase) through MAPK-interacting kinase 1 and 2 (Mnk1/2), mitogen- and stress-activated protein kinase 1 (Msk1), and MAPK-activated kinase 2/3 (MK2/3), respectively, which is thought to enhance translation of some mRNAs [36], [37], [38], [39]. On the other hand, Nrf2 has been shown to be regulated at the translational level under several conditions [40], [41]. Thus, MAPKs-mediated indirect control of Nrf2 protein synthesis, along with the moderate role of direct phosphorylation on Nrf2, may represent the underlying mechanism by which MAPKs regulates the Nrf2 signaling pathway.

## Supporting Information

Figure S1 HEK293T cells were co-transfected with expression vectors for HA-Nrf2 wild-type or mutants and different kinases. Cell lysates were collected in denaturing conditions, and phosphorylation analysis was performed as described in Fig. 1A and 3A. All three sites (S215, S408 and S577) contribute to the overall phosphorylation level of Nrf2 in the presence of all four kinases.
(0.81 MB TIF)

Figure S2 HEK293T cells were co-transfected with expression vectors for NF-κB p65, along upstream kinases IKKα and IKKβ in HEK293T cells. Phosphorylation of p65 at S536 by IKKs was confirmed by an antibody that recognizes this site-specific modification (Cell Signaling, Cat# 3033S). The antibody for phospho-S/TP did not pick up any signal above background, indicating the specificity of this antibody.
(0.83 MB TIF)
