# Supplementary figures and images for: Phosphorylation of Nrf2 at Multiple Sites by MAP Kinases Has a Limited Contribution in Modulating the Nrf2-Dependent Antioxidant Response
Source: PLoS One. 2009 Aug 11;4(8):e6588. doi: 10.1371/journal.pone.0006588 (PMC2719090; doi:10.1371/journal.pone.0006588)

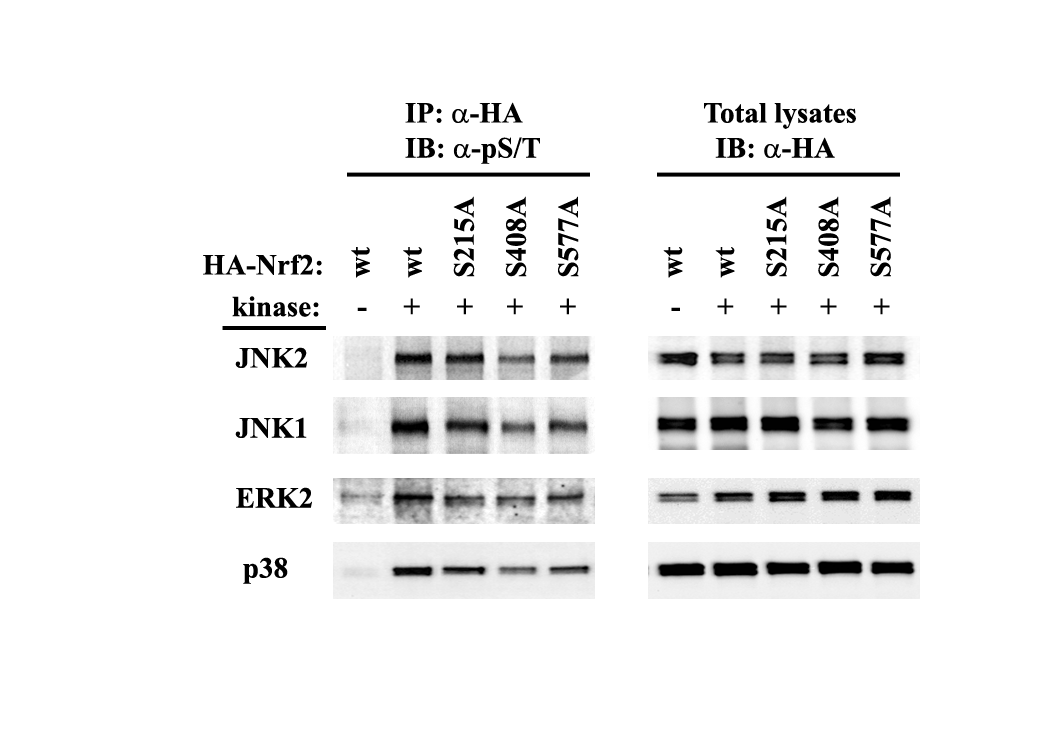

Supplement: Figure S1 — HEK293T cells were co-transfected with expression vectors for HA-Nrf2 wild-type or mutants and different kinases. Cell lysates were collected in denaturing conditions, and phosphorylation analysis was performed as described in Fig. 1A and 3A. All three sites (S215, S408 and S577) contribute to the overall phosphorylation level of Nrf2 in the presence of all four kinases. (0.81 MB TIF) [file pone.0006588.s001.tif]

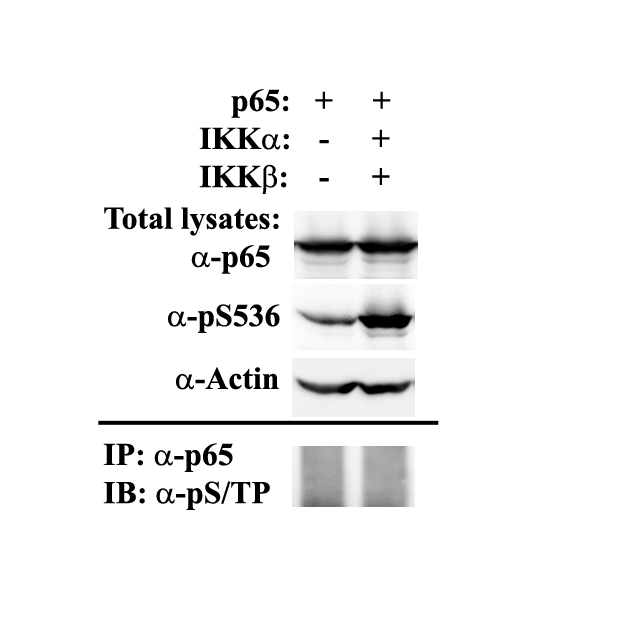

Supplement: Figure S2 — HEK293T cells were co-transfected with expression vectors for NF-κB p65, along upstream kinases IKKα and IKKβ in HEK293T cells. Phosphorylation of p65 at S536 by IKKs was confirmed by an antibody that recognizes this site-specific modification (Cell Signaling, Cat# 3033S). The antibody for phospho-S/TP did not pick up any signal above background, indicating the specificity of this antibody. (0.83 MB TIF) [file pone.0006588.s002.tif]
